# Therapeutic Response in Patients with Advanced Malignancies Treated with Combined Dendritic Cell–Activated T Cell Based Immunotherapy and Intensity–Modulated Radiotherapy

**DOI:** 10.3390/cancers3022223

**Published:** 2011-04-28

**Authors:** Kenichiro Hasumi, Yukimasa Aoki, Ryuko Watanabe, Kim G. Hankey, Dean L. Mann

**Affiliations:** 1 Hasumi International Research Foundation, Tokyo Research Center, 1-44-6 Asagaya-kita, Suginami- ku, Tokyo 166-0001, Japan; E-Mails: kenhasumi@aol.com (K.H.); aoki@cccc-sc.jp (Y.A.); watanabe@shukokai.org (R.W.); 2 Department of Pathology, University of Maryland School of Medicine, MSTF Room 700, 10 South Pine Street, Baltimore, Maryland 21040, USA; E-Mail: khank001@umaryland.edu (K.G.H.)

**Keywords:** dendritic cells, activated T-cells, combination immunotherapy, intratumoral injection, cancer vaccine

## Abstract

Successful cancer immunotherapy is confounded by the magnitude of the tumor burden and the presence of immunoregulatory elements that suppress an immune response. To approach these issues, 26 patients with advanced treatment refractory cancer were enrolled in a safety/feasibility study wherein a conventional treatment modality, intensity modulated radiotherapy (IMRT), was combined with dendritic cell-based immunotherapy. We hypothesized that radiation would lower the tumor burdens, decrease the number/function of regulatory cells in the tumor environment, and release products of tumor cells that could be acquired by intratumoral injected immature dendritic cells (iDC). Metastatic lesions identified by CT (computed tomography) were injected with autologous iDC combined with a cytokine-based adjuvant and KLH (keyhole limpet hemocyanin), followed 24 h later by IV-infused T-cells expanded with anti-CD3 and IL-2 (AT). After three to five days, each of the injected lesions was treated with fractionated doses of IMRT followed by another injection of intratumoral iDC and IV-infused AT. No toxicity was observed with cell infusion while radiation-related toxicity was observed in seven patients. Five patients had progressive disease, eight demonstrated complete resolution at treated sites but developed recurrent disease at other sites, and 13 showed complete response at various follow-up times with an overall estimated Kaplan-Meier disease-free survival of 345 days. Most patients developed KLH antibodies supporting our hypothesis that the co-injected iDC are functional with the capacity to acquire antigens from their environment and generate an adaptive immune response. These results demonstrate the safety and effectiveness of this multimodality strategy combining immunotherapy and IMRT in patients with advanced malignancies.

## 1. Introduction

There are few treatment options for cancer patients who failed conventional therapies and have progressive disease. Often, the therapeutic modalities offered consist of phase 1 to 2 trials designed to assess toxicity of new or alternate combinations of therapeutic agents and to determine potential efficacy. Over the past decade there has been an increasing interest in the application of immunotherapeutic approaches to treat cancers; indeed, many such trials have been conducted in patients with advanced disease [1-3]. In most instances, the approaches have been directed at employing the cellular arm of the immune response by infusion of large numbers of effector cells generated by *ex-vivo* expansion of peripheral blood or tumor infiltrating lymphocytes or by vaccination with the intent of inducing anti-tumor cellular immunity [4,5]. In a number of trials, dendritic cells have been used as the vector for vaccine delivery [1].

Dendritic cells (DC) are the sentinel antigen presenting cells in the body [6]. DC are progeny of the myelomonocytic cell lineage and are found in areas of the body that are commonly exposed to environmental pathogens where they acquire potentially antigenic materials, process these substances, and migrate to secondary lymphoid structures where immune responses to the antigens and self-constituents are generated. This cascade of events is regulated by a battery of cytokines and chemokines that are produced by DC upon activation and by non-lymphoid cells that are responding to environmental “danger signals” [7]. Additional cytokines and chemokines are produced by both DC and lymphoid cells as they interact during the process of antigen presentation. During this process DC induce effector as well as regulatory immune responses, the latter a critical consideration in any attempt to induce effective immune responses to tumor-associated antigens (TAA) [8-10].

DC-based cancer vaccines have been prepared by *in vitro* differentiation of peripheral blood monocytes into immature DC (iDC) using a variety of cytokines including GM-CSF and IL-4 [11]. The iDC are loaded with TAA by a variety of methods including: (1) exposure to proteins expressed by tumors, HLA-restricted peptide constituents of TAA, and lysed autologous or allogeneic tumor cells; (2) electroporation-based delivery of DNA encoding constituents of TAA or autologous tumor cell mRNA; and (3) lipofection with TAA. The efficiency of antigen presentation may be enhanced by exposing the antigen loaded iDC to cytokines that mature the DC prior to their administration and/or by co-administration of adjuvants such as vectors that produce cytokines or substances that activate DC through Toll-like receptors [1,12-14]. The route of administration of DC-based vaccines varies and includes injecting iDC directly into tumors where they can acquire products of tumor cells and initiate immune responses to the resident cancers [13,15-22].

There is evidence that vaccines directed toward TAA and delivered by DC induce cellular immune responses in humans as measured *in-vitro* using peripheral blood lymphocytes. However, there is limited evidence that these responses translate into clinical benefit with the possible exception of the short increase in survival of advanced prostate cancer patients who received Provenge, an autologous DC-based vaccine [23,24]. There are a number of reasons for the lack of clinical response as manifested by tumor regression in patients that develop demonstrable immunity to cancer vaccines. One issue is the immunoregulatory environment present at the site of intended cytolytic activity [8]. A host of studies have clearly demonstrated the presence of regulatory T-cells and myeloid-derived suppressor cells in tumors [25-27]. In addition, cytokines produced by various cells in the tumor are known to down-regulate both afferent and efferent arms of the immune response [28,29]. Once effector cells are generated, they may be released into circulation but fail to reach the targeted site due to lack of a tumor-produced chemokine gradient and/or surface expression of complimentary chemokine receptors that together regulate trafficking of effector cells [30]. Another overriding issue is the lack of sufficient numbers of effector cells that would be required to kill large numbers of malignant cells, a likely scenario in patients who failed prior therapy. Thus immunotherapy may only be effective when the tumor burden is low and the immunoregulatory responses are themselves suppressed.

One approach that may address the above issues is to combine immunotherapy with other therapeutic measures with the intent to decrease the tumor burden while controlling immunoregulatory cells in the tumor environment [31-33]. Several approaches have been explored in animal models. These include administration of chemotherapeutic agents prior to vaccination and treatment of the tumor with local hyperthermia or radiation followed by intratumoral injection of iDC [15,34-38]. Radiation not only alters the immunoregulatory environment but also causes cell death, releasing potential antigenic constituents of the tumor cell and inducing an inflammatory response with production and release of cytokines that support and enhance the function of DC [16,17,20,38,39].

We previously reported the lack of adverse reactions in advanced cancer patients treated with intratumoral injection of iDC alone or in combination with chemotherapy or local radiotherapy [40]. This observation, together with the background data reviewed and summarized above, establishes the rationale for the testable hypothesis that combining intratumoral injection of iDC with local irradiation to the injected site is an effective treatment modality for patients with advanced cancers. Herein we report the safety and feasibility of this approach in a trial where this hypothesis is examined.

## 2. Materials and Methods

### 2.1. Patients

Patients enrolled in this trial were self-referred to either the Shukokia Clinic or the Tokyo Clinic and Research Institute. All patients had recurrent or stage IV malignancies having failed prior standard surgical and/or adjuvant therapy. Additional enrollment criteria included the presence of tumors that measured 3 cm or less and a minimum lapse of three months from prior therapy to protocol enrollment. Patients gave written consent to the procedures and protocols after detailed explanation and discussion. The consent forms and procedures were approved by the Institutional Review Boards of the respective institutions.

### 2.2. Treatment Protocol

**Figure 1.**
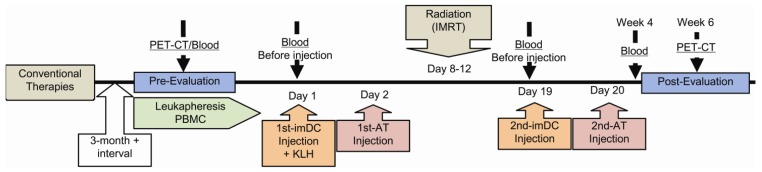
Sequential events in the treatment protocol.

Figure 1 illustrates an overview of the treatment protocol. Prior to treatment, the extent of disease and the location of metastases were established by PET-CT (Positron Emission Tomography—Computed Tomography). The patients underwent leukapheresis to obtain monocytes for differentiation into iDC and a monocyte depleted T-cell enriched population for preparation of activated T-cells (AT). After preparation of these reagents, a portion of the iDC was combined with lymphocyte conditioned media, a multi-cytokine based adjuvant (LCM), and KLH. This cell mixture was equally divided based on the number of sites to be injected, the volume of each aliquot was adjusted to ∼2 mL with PBS, and the individual lesions were injected under CT guidance. AT were infused the following day. Approximately 3 days later, the injected tumor sites received Intensity-Modulated Radiotherapy (IMRT) in divided doses. Two to three days following the last dose of radiation, the tumor sites were again injected with iDC suspended in LCM (without KLH) and AT infused the next day. Blood samples were obtained prior to protocol initiation and periodically thereafter to monitor serum levels of tumor markers and the development of anti-KLH antibodies. The PET-CT exams were repeated 6 weeks after the last treatment and periodically thereafter. The treatment cycle was repeated for several patients who developed lesions at new sites.

### 2.3. Collection and Isolation of Peripheral Blood Mononuclear Cells (PBMCs)

Leukaphereses were performed on a COBE Spectra blood separator (Gambro KK, Tokyo, Japan) using the program for collection of the mononuclear cell population (MNC) (version 7.1). MNC were further purified by density gradient centrifugation, the cells washed, and a portion used to isolate monocytes for differentiation into iDC and to prepare AT. Remaining MNC were cryopreserved in AIMV media (Invitrogen Gibco, Tokyo, Japan) containing 10% DMSO and stored in vapor phase of liquid nitrogen. To prepare iDC and AT from cryopreserved MNC, the MNC were thawed in a 37 °C water bath and washed twice in AIMV medium. iDC and AT were prepared as described below.

### 2.4. Preparation of Immature Dendritic Cells

iDC were differentiated from monocytes by the loosely adherent method as previously described [40]. In brief, PBMC (∼6 × 10^8^) were suspended in 20 mL AIMV media and distributed to four T75 cm^2^ polystyrene flasks containing 10 mL of AIMV media. After two hours at 37 °C, non-adherent cells were removed, transferred to conical tubes, and reserved for AT preparation (see below). Fifteen milliliters of DC growth medium consisting of AIMV medium supplemented with 800 IU/mL GM-CSF and 500 U/mL IL-4 (CellGenix, Germany) was added to each flask containing adherent cells. Flasks were incubated at 37 °C with 5% CO_2_ and supplemented with an equal volume of growth media on day 3. The iDCs were harvested on day 7 and either prepared for injection or cryopreserved in AIMV medium containing 20% autologous serum and 10% DMSO.

### 2.5. Preparation of Activated T-cells [40]

Approximately 6–9 × 10^8^ non-adherent cells obtained from the monocyte isolation described above were suspended in 20 mL of AIMV medium containing 10% autologous serum and supplemented with 1000 IU/mL IL-2 (Proleukin, Novartis, Emeryville, CA) and distributed into 4 flasks pre-treated with 10 mL of PBS containing 5 mcg/mL anti-CD3 (Orthoclone OKT3 injection, Janssen Pharmaceutical KK, Japan). Flasks were incubated for 7 days at 37 °C with 5% CO_2_ and supplemented with an equal volume of media at day 3 or 4. Three hours prior to harvesting, 1 mcg/mL ionomycin (Sigma, St. Louis, MO) was added to the medium. The cells were harvested, washed three times, and portions prepared either for infusion or cryopreservation as described above.

### 2.6. Preparation of LCM

Preparation of products of activated lymphocytes that were used as an adjuvant in these protocols and the cytokine/chemokine content of the adjuvant are detailed elsewhere [41]. In brief, lymphocytes were suspended in 50 mL XVIVO 10 medium (Cambrex, Walkersville, MD) containing human T-expander CD3/CD28 Dynabeads (Invitrogen Dynal AS, Oslo, Norway) at 1 cell to 1 bead ratio. The combination was incubated at 37 °C in 5% CO_2_ for 2 days. Cytokine-rich supernatants were harvested by centrifugation at 300 × g for 7 min and stored at 4 °C for later use. Sterility and endotoxin testing were carried out as described below.

### 2.7. Sterility and Endotoxin Testing

Seven days prior to cell harvest, presence of microbial contaminants was tested by incubation of cultured cells on agar at 37 °C with subsequent inspection for bacterial growth. Endotoxin levels (<0.5 EU/mL) were determined using a commercially available chromogenic endotoxin assay kit according to the manufacturer's instruction (Toxicolor system LS-50M, Seikagaku Corp., Tokyo, Japan). Only those cultures with a negative test result were administered.

### 2.8. Characterization of DC and AT

A standard flow cytometry labeling protocol was used to determine cell surface marker expression on iDCs using fluorochrome-conjugated monoclonal antibodies to CD11c, CD14, CD40, CD80, CD83, CD86 and HLA-DR (BD Pharmingen, Japan). AT were evaluated for expression of CD3, CD4, CD8, CD11c, CD14, CD19, CD25, CD45, CD56, CD154, and HLA-DR following culture. Minimally 5,000 events were acquired on a BD FACSCalibur (BD Biosciences, Japan) and data were analyzed using Cell Quest analysis software. The phenotype of the injected iDC and infused AT is summarized in Table 1.

### 2.9. Preparation of Cells for Injection

iDC and AT were thawed in a 37 °C water bath approximately 1 hour prior to planned injection. One milliliter of AIMV media was added to each thawed vial, suspensions were incubated for 2 min at 22 ± 3 °C (room temperature, RT) and cells were transferred into 50 mL of media and centrifuged at 300 × g for 7 min to remove DMSO. Cells were resuspended in fresh media, counted, and sterility and endotoxin testing samples removed. Remaining iDC were suspended in PBS containing 10% LCM. Based on the number of sites to be injected, the iDC were distributed into two or more microtubes and placed on ice for transport to the clinic where they were injection into the patient's metastatic lesions. AT were suspended in 100 mL of normal saline and infused IV over a period of 30–40 min.

### 2.10. Radiation Therapy

Intensity Modulated Radiotherapy was delivered to each site injected with iDC. The appropriate prescribed total dose was determined to be such that the biologically effective dose was 72 Gy according to the linear quadratic model. The fraction size was optimized such that the estimated radiation induced late toxicity of surrounding normal tissue would be equal to or less than grade 2 based on the NCI-CTC version 2.0 scaling system.

### 2.11. Toxicity Monitoring

Patients were monitored for adverse reactions to therapy. Indices checked include vital signs (temperature, blood pressure, pulse, respiration), blood chemistry and hematology, diaphoresis, arthralgia, and pain and/or swelling at the injection site. Other signs and symptoms that might be associated with radiation or the injection of cells by insertion of a needle through various organs in order to reach metastatic lesions that are present in body cavities (*i.e.*, peri-aortic lymph nodes) were monitored.

### 2.12. Evaluation of Response

PET-CT imaging was used to assess response to treatment following RECIST criteria. The first exam was conducted ∼6 weeks after the end of the last treatment cycle with periodic follow-up CT or PET-CT exams. Complete response (CR) was defined as the resolution of the treated site and no new lesions at distant sites at follow-up with radiographic exams. Patients who had an initial complete response at the treated sites at the 6-week CT exam but developed radiographic evidence of disease on subsequent evaluation were designated as having recurrent disease (RD) even though they experienced a disease-free interval. Finally, progressive disease (PD) was defined as no appreciable diminution in the size of the treated lesion and/or the appearance of new lesions at first follow-up PET-CT exam.

### 2.13. Detection of Anti-KLH or Anti-Mesothelin Antibodies

Serum antibodies specific for KLH and mesothelin were detected by sandwich ELISA. Test wells were coated with 1.0 mcg/mL KLH (Calbiochem, San Diego, CA) in 1 × PBS or 20 mcg/mL mesothelin lysate (Novus Biologicals) in 0.1 M carbonate buffer. For KLH ELISA, control wells were treated with 1 × PBS only. Plates were incubated overnight at 4 °C, washed with PBST (PBS + 0.05% Tween 20), and blocked with 200 μL of 5% non-fat dried milk/PBST for 2 h at RT. After additional washing, 100 μL of serum diluted in blocking buffer was added in triplicate, plates were incubated at RT for 1 h, washed, and incubated for another hour with 100 μL of peroxidase-conjugated goat anti-human IgG (KPL, Gaithersburg, MD) diluted to 1:30,000 for KLH or 1:1,000 for mesothelin. After washing, assays were developed with 100 μL/well of SureBlue TMB Peroxidase Substrate (KPL) for 30 min at RT and terminated by addition of 1 N HCl. Optical density was measured at 450 nm using a VersaMax ELISA reader (Molecular Devices, Sunnyvale, CA). OD_450nm_ of specific anti-KLH antibodies was determined by subtracting the mean OD for wells coated with PBS alone (background) from mean OD obtained in wells coated with KLH. For anti-mesothelin ELISA, data was reported as percent increase in OD_450nm_ units by comparing the reactions of sera collected before and after treatment.

### 2.14. Quantitation of Cytokine Response to Tumor Lysate

PBMC collected from a breast cancer patient at enrollment and during treatment were stimulated with 10 mcg/mL freeze-thaw cell lysates of the breast cancer cell line, MCF7. Cells were cultured at 10^6^ cells/mL in 96-well trays for 48 h. Cell culture supernatants were harvested and analyzed for cytokine content by Luminex 100 multiple cytokine detection assay following the manufacturer's instruction (Luminex 100, Austin, TX).

## 3. Results

### 3.1. Patients

Patient demographics, diagnosis, and an overview of past treatment information are shown in Table 2. The distribution of cancers treated on this protocol reflects the incidence of the different cancers in Japan. In addition, the distribution of patient age is typical for patients with the different cancers who had received and failed prior therapy. The majority of patients were treated with surgery at some time during the course of their disease and all patients received adjuvant therapy consisting of either radiation or chemotherapy, or both.

### 3.2. Treatment and Toxicity

A summary of the extent of patients' diseases, number of metastatic lesions observed radiographically, doses of iDC and AT, IMRT dosages and periodicity of delivery, treatment-related toxicity and outcome is provided in Table 3. There is considerable variation amongst the cohort with respect to the scope of existing disease at the time of enrollment, as evidenced by the number of sites identified by PET-CT. The listed sites of recurrence represent the reappearance of cancer at or near the location of primary disease as well as multiple metastases at various anatomic locations. For each patient, the total number of iDC injected was dependent on the number recovered from the cultures. The cells were equally divided amongst their identified sites of metastasis and local recurrence. The number of AT infused varied according to the number of cells recovered from the cultures. Each of the metastatic sites was treated with fractionated doses of IMRT followed by re-injection of iDC at these sites and infusion of AT. Importantly, there were no complications related to the intratumoral injection of iDC and no toxicity related to intravenous infusion of AT. Radiation associated toxicities were observed in seven patients and do not appear to be related to tumor type, location of tumor, radiation dose, or number of fractions delivered.

### 3.3. Response

Therapeutic response was evaluated by PET-CT or CT at 6 weeks after the last treatment and periodically thereafter. The patients on this study are listed in Table 3 ordered by days of disease-free survival. At the 6-week evaluation, five of 26 patients had progressive disease while the remaining patients exhibited complete response at the treatment site. Of these 21 patients, eight were found on subsequent follow up to have recurrent disease at sites distant from that of the original treatment. The remaining thirteen patients had complete responses over the duration of their evaluation period. The estimated restricted mean disease-free time in the 21 patients who had initial response is 345 days and the estimated median is 377 days.

**Figure 2.**
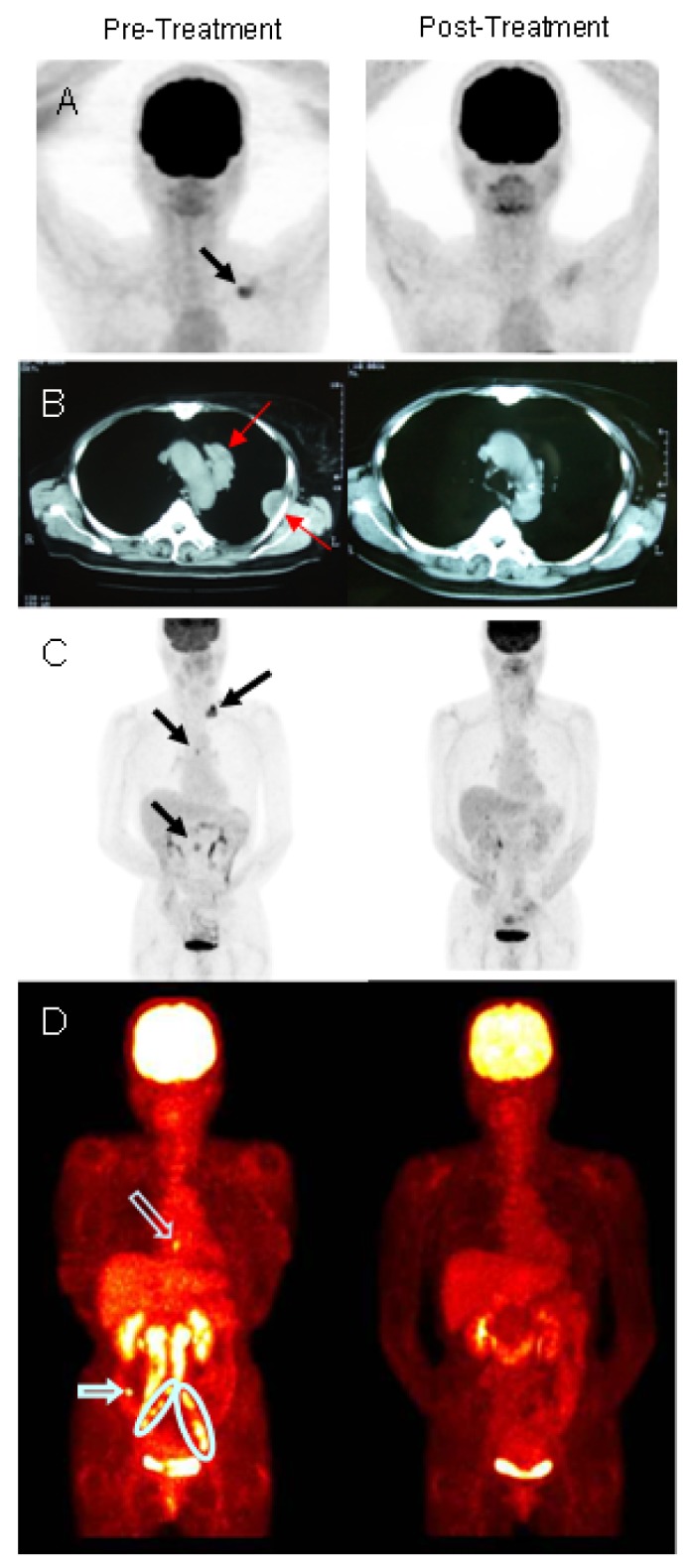
Radiographic evidence of response to treatment. (**A–C**). Computed tomography (CT) radiographs showing metastatic cancer sites (solid arrows) before and after treatment in two breast cancer and one gastric cancer patient, respectively; (**D**). PET-CT showing resolution of treated (circled) and un-treated metastatic sites (open arrows) in a patient with cervical cancer. Circled sites were injected with immature dendritic cells (iDC) followed by intensity modulated radiotherapy (IMRT) as per protocol and resolved as were untreated metastatic lesions (open arrows).

Examples of radiographic evidence of response to treatment are illustrated in Figure 2. Panel A shows the pre- and post-treatment CT of a breast cancer patient with metastatic disease confined to the supraclavicular region, probably lymph nodes. Another breast cancer patient had a complete response when treated for metastatic breast cancer involving the mediastinum and the chest wall (Figure 2B). The radiographic images of a patient with stomach cancer (Figure 2C) illustrate multiple metastatic lesions in the supraclavicular region, mediastinum, and periaortic lymph nodes at enrollment that completely responded to treatment. Figure 2D illustrates resolution of metastatic tumors at sites distant from the areas receiving iDC injection and IMRT in a patient with cervical cancer.

### 3.4. Serum Tumor Markers

**Figure 3.**
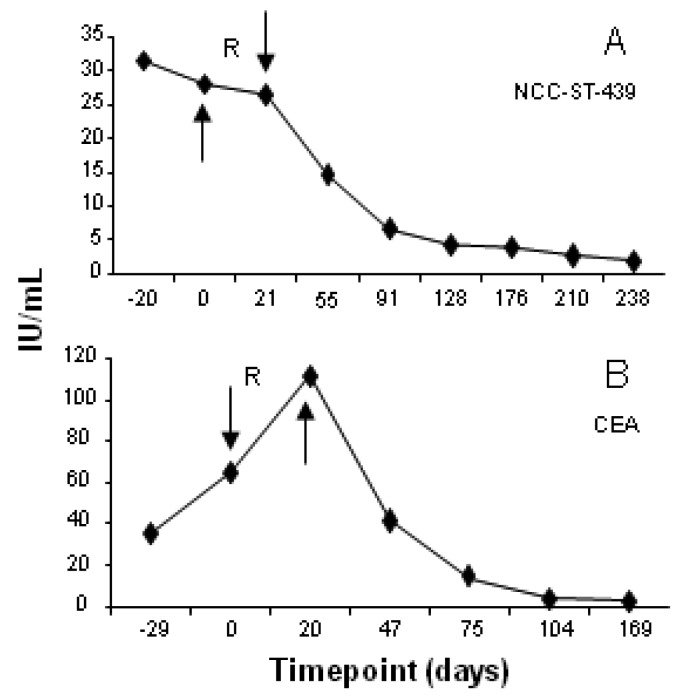
Serum levels of tumor markers relative to treatment sequence. Arrows indicate timepoints of intratumoral injection of iDC and infusion of AT; R denotes the receipt of IMRT. (**A**). Elevated levels of NCC-ST-439 declined to normal after treatment in serum samples obtained from a patient with metastatic breast cancer; (**B**). Elevated serum levels of CEA fell to normal after treatment in a patient with metastatic GI cancer.

In addition to radiographic studies, serum tumor markers were used to monitor treatment response. Representative results are shown in Figure 3. NCC-ST-439 levels declined after treatment in serum samples obtained from a patient with metastatic breast cancer (Figure 3A) while serum CEA levels fell to normal after treatment and remained at that level in the follow-up period for a patient with metastatic gastric carcinoma (Figure 3B).

The results of tumor marker studies in patients where markers of disease are generally accepted to be informative are summarized in Table 4. The values shown in this table were obtained in serum samples drawn at initial evaluation (Pre), at the first or second evaluation after completion of iDC injections and AT infusions (Post-1), and at the latest post-treatment evaluation (Post-2). Serum tumor marker levels decreased in post-treatment samples of all patients tested, returning to normal in seven of the 11 patients indicating that tumor load was significantly decreased with the treatment process.

### 3.5. KLH Antibody Response

**Figure 4.**
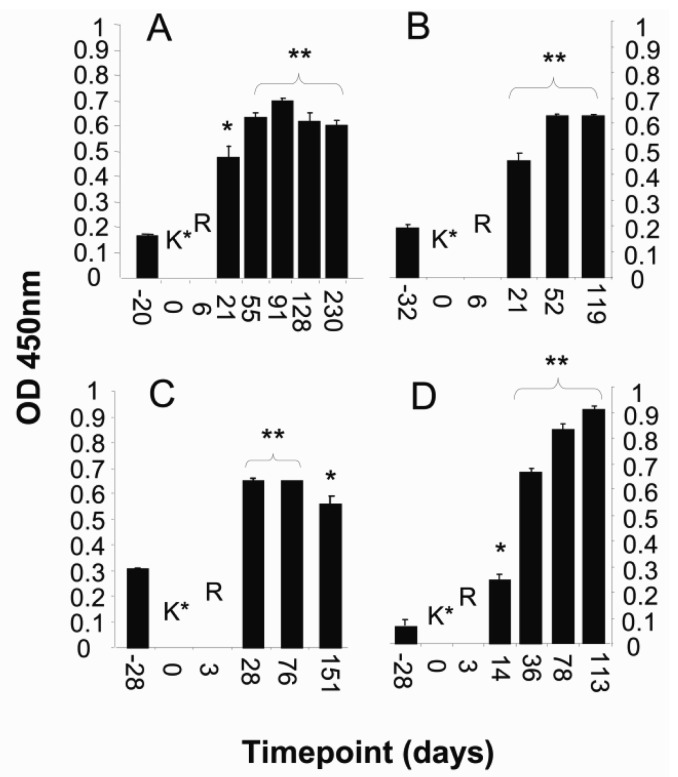
Serum levels of anti-KLH antibody relative to treatment sequence. Representative data from patients with cancer of the (**A**) breast; (**B**) ovary; (**C**) stomach; and (**D**) lung. Anti-KLH assessed by ELISA in serum samples obtained before and after treatment. KLH administration occurred at timepoint 0 and is denoted by K*; R indicates the time at which the immunization sites were irradiated. Significant increases in anti-KLH antibody levels following treatment are indicated by double (*p* < 0.005) and single (*p* < 0.05) star.

KLH was co-administered with the initial iDC injections as a marker to investigate the ability of iDC to acquire antigen from their environment and generate an immune response. Twenty-three of the 26 patients were immunized with 1mg of KLH combined with the first iDC and LCM preparation that was subsequently apportioned according to the number of sites to be injected. Serum or plasma samples obtained prior to treatment and at periodic intervals thereafter were tested for antibodies to KLH. Anti-KLH antibodies were detected in 16 of the 23 vaccinated patients (data not shown). The kinetics of antibody response in four representative patients is illustrated in Figure 4.

**Figure 5.**
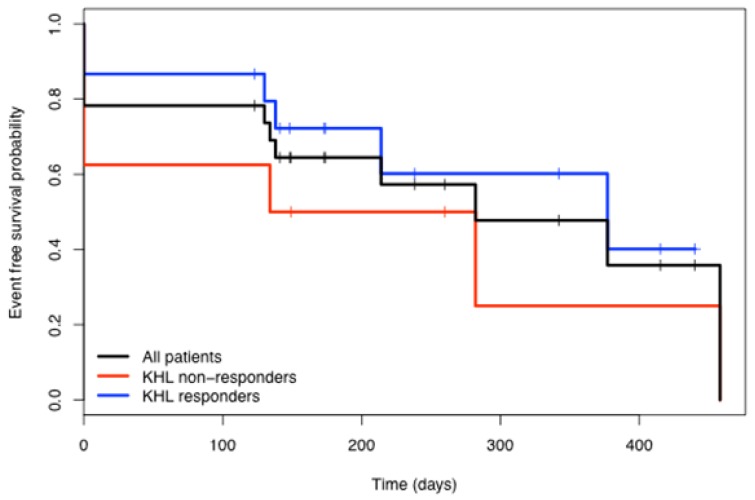
Kaplan-Meier analysis comparing the number of disease-free days of all patients with those that did or did not develop anti-KLH antibodies.

Interestingly, the frequency of KLH immunity was higher in the complete response group (81.82%) than in the combined recurrent and progressive disease groups (58.33%). To determine if KLH responses were correlated with response to therapy, Kaplan-Meier analysis of disease-free survival of the two groups were compared (Figure 5). The data suggest that the patients who responded to KLH immunization had a better overall survival. However, the difference was not statistically significant (p = 0.31) when analyzed using the exact log-rank test from StatXact.

### 3.6. Immune Response to Tumor Antigens

**Figure 6.**
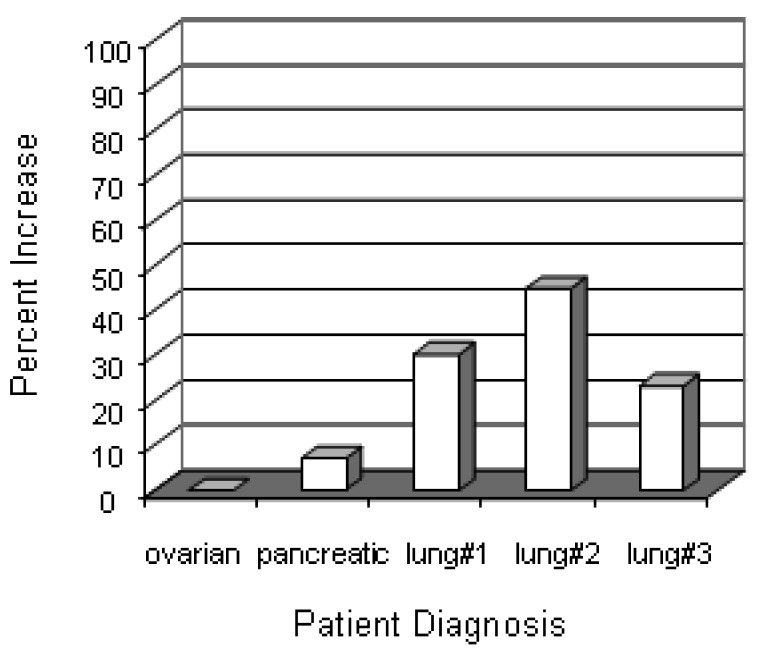
Serum levels of anti-mesothelin antibody increases after treatment. Pre- and post-treatment sera were collected from 5 patients (3 lung cancer, 1 ovarian, 1 pancreatic) and screened for antibodies against mesothelin by ELISA. Data represent the percent increase in OD_450nm_ units of post-treatment sera over the corresponding pre-treatment sera values.

We have begun to investigate immune responses to antigens that may be overexpressed by tumors of different histological origin. Specifically, we questioned whether treatment induced a humoral response to mesothelin, a protein that has been shown to be overexpressed in pancreatic, gastric, ovarian, and lung carcinoma. Our preliminary results are presented in Figure 6 where post-treatment sera from three lung cancer patients showed an increase in anti-mesothelin antibody titer above the titer detected in sera obtained before therapy. Sera from two patients with ovarian and pancreatic cancer showed no changes.

Using an alternate approach, we exposed PBMC from a patient with breast cancer to lysates from the breast cancer cell line, MCF-7, and measured cytokine production as a criteria of antigen recognition and response. As shown in Table 5, there was a significant increase in the amount of IL-10, IL-1β, IL-6 and TNFα produced by cells obtained 25 days post-treatment compared to cells procured at enrollment and after therapy. Interestingly, levels of IL-2, IFNγ, and IL-17 did not increase upon exposure to lysates. We speculate but have no proof that the response observed is due to recognition of antigenic determinants shared by the MCF-7 cell line and the patient's tumor.

## 4. Discussion

We investigated the safety and feasibility of a therapeutic approach combining immunotherapy with radiation to treat patients who developed recurrent cancer in spite of a history of extensive treatment with standard modalities. We hypothesized that this multimodality treatment approach is not only safe but also potentially effective for treating patients with recurrent and/or metastatic cancers. The rationale for this study was based on the results of preclinical studies conducted by ourselves and others that demonstrate the potential therapeutic efficacy of combining immunotherapy with radiation to treat cancers.

The immunotherapeutic component of the protocol was derived from the extensive body of knowledge regarding the fundamental role of DC in innate and adaptive immunity and their application to cancer therapy. The DC used in this protocol were differentiated from autologous peripheral blood monocytes in media containing GM-CSF and IL-4 to become immature DC, a state of maturation that is considered optimal for antigen acquisition [42,43]. To arm the iDC with products of the tumors, iDC were injected directly into metastatic and recurrent tumor sites. The rationale for this approach is supported by studies in animal models and in several clinical trials that have documented the capacity of iDC to generate immune responses to TAA when injected directly into a tumor [13,15-18,20-22,44].

Cytokines and chemokines present in the iDC environment play a fundamental role in the generation of immune responses to acquired antigens and control or dictate the type and character of the T-cell response (effector *versus* suppressor). There is abundant evidence that immunosuppressive cytokines are constitutively present in the tumor microenvironment. Radiation, as employed in this protocol, has been shown to produce danger signals accompanied by production of cytokines and chemokines that favor an effector immune response [39]. In addition to this radiation induced response, cytokine and chemokine products of *in-vitro* activated T-cells (designated LCM) were injected with the iDC into the tumor site. We previously documented the adjuvant-like properties of LCM, showing that this combination of cytokines and chemokines caused differentiation of monocytes into immature and mature DC, enhanced *in-vitro* immune responses to primary and recall antigens, and augmented antibody and T-cell responses *in-vivo* when administered with several standard vaccines to non-human primates [41].

Intratumoral iDC injection was followed with IV infusion of AT-cells some 24 h later, an immunotherapeutic approach that has also been employed by others to treat cancer [22]. AT-cells are known to produce cytokines that support the development of an adaptive immune response. Furthermore, AT express CD40L, another molecule with the potential to trigger maturation of the injected iDC. The IV-infused AT contain a subset of cells expressing surface markers for NK and NKT, cells known to have cytolytic properties (Table 1).

IMRT was delivered to each of the recurrent and metastatic tumor sites that had been injected with iDC. The combination of radiotherapy with immunotherapy has been proposed as a potential cancer treatment by a number of investigators and indeed applied in animal models and several clinical trials [16,17,22]. In addition to generating danger signals and release of soluble mediators that support and augment innate and adaptive immune response, radiation has been shown to induce apoptosis and necrosis of tumors with concomitant release of tumor-derived products. These products (TAA) can be acquired by DC that, under the influence of the appropriate cytokine environment, migrate to the secondary lymphoid organs where adaptive immune responses are generated. The importance of IMRT as the radiation delivery modality in this protocol should be noted [45]. A major concern in radiation treatment of cancers is the collateral damage to non-involved adjacent tissues. Conventional radiation to multiple sites in various anatomic locations may give rise to unwanted toxicity to critical structures and organs adjacent to the treatment sites. IMRT has been shown to reduce the dose of radiation to structures in the immediate vicinity of the treated site [45,46]. Indeed, only 7 of the 26 patients enrolled experience radiation-related toxicity (Table 3). No demonstrable toxicity related to the immunotherapy component of this trial was noted.

KLH has been used to assess immune responses in immunotherapeutic trials employing DC-based vectors for delivery of cancer vaccines [47,48]. In our protocol KLH was co-injected with iDC to monitor the capacity of the iDC to acquire antigenic substances from their environment when injected into the metastatic or recurrent tumors. Sixteen of 23 patients developed antibodies to KLH as detected in serum samples obtained at various times after immunization. These results indicate that the DCs, either those injected and/or in residence, were not only capable of acquiring antigen from the tumor environment but were also capable of migrating from that location to regional lymphoid organs where an adaptive immune response was generated. Interestingly, radiation to the site of injection did not abrogate the development of KLH antibodies suggesting that the DCs were resistant to effects of radiation or—more likely—they had already migrated to secondary lymphoid organs prior to IMRT. The KLH response appeared to be more frequent in patients who responded to treatment; however, this difference was not statistically significant (Figure 5).

There were two components of the protocol that have therapeutic potential; that is, the immunologic response to the tumor and the effect of radiation. It is difficult, perhaps even impossible, to ascribe the responses observed to one or the other component. There are, however, several lines of evidence that we feel support our hypothesis that the combination employed is complimentary. The most robust argument is illustrated by the PET-CT shown in Figure 2D that clearly demonstrates the regression of metastatic lesions that were not subjected to either radiation or direct injection with iDC after such treatment of other lesions. Similar responses were seen in other patients.

Development of an adaptive immune response to constituents of the tumor would be most likely responsible for destruction of untreated metastases. The adaptive immune response to KLH demonstrates the capacity of the immunotherapeutic component of this protocol to generate an immune response to an environmental–albeit surrogate–antigen. We have begun a systematic investigation of immune responses to tumor antigens that might be expressed by the patients' cancers. The results of some preliminary tests are shown in Table 5 and Figure 6.

We acknowledge that this limited data does not support an argument that the observed responses are due to both an immunologic and radiation effect. However, since the objective of any cancer therapy is directed at the well being of the patient, we feel that the combination approach employing these modalities has benefited these patients by objectively reducing their demonstrable tumor burden with limited side effects and toxicity.

## 5. Conclusions

In summary, combining immunotherapy with radiation was shown to successfully eliminate metastatic and recurrent tumors on initial treatment in 21 of 26 patients with 13 of the 26 having no evidence of recurrent disease when evaluated by CT (or PET-CT) at various intervals of follow-up. The overall disease-free interval of the responding patients at the current time is 377 days. This remarkable response supports the concept that combinations of conventional anti-cancer therapies and cancer immunotherapy are worthy of investigation in patients with advanced cancers as well as in patients that are undergoing primary adjunctive therapy for their disease.

## Figures and Tables

**Table 1.** Phenotypic characteristics of cell preparations.

**Dendritic Cells**		**Activated T-cells**	
Marker	Percentage	Marker	Percentage
CD11c	87 ± 12	CD3^+^CD4^+^	51 ± 17
CD14	32 ± 28	CD3^+^CD8^+^	38 ± 14
HLA-DR	69 ± 23	CD3^+^CD56^+^	31 ± 16
CD40	39 ± 27	CD3^-^CD56^+^	8 ± 5
CD80	30 ± 18	CD62L	16 ± 7
CD83	22 ± 15	CD154	25 ± 13
CD86	70 ± 23	CD25	88 ± 18
CD3	3 ± 3		

**Table 2.** Patient demographics, clinical diagnosis, and prior treatment.

**Cancer Diagnosis**	**Number of Evaluable Patients**	**Age (years)**		**Prior Therapy Number of Patients**	
		**Median**	**Range**	**Surgery**	**Adjuvant**
Breast	6	55	45–68	6	6
Cervical/Uterine	3	64	40–65	3	3
GI	6	72.5	62–83	6	6
Lung	4	64	54–83	1	3
Lymphoma	1	57	--	1	1
Ovarian	2	49.5	47–52	2	2
Pancreatic	2	78	74–82	0	2
Prostate	1	69	--	0	1
Renal	1	66	--	1	1

**Table 3.** Treatment and clinical response.

Cancer	Sites of Tumor Recurrence and Treatment^1^	Total # of cells Injected,		IMRT		Total # of cells Injected,		Disease-Free Follow-up (days)	Treatment Response 2	Toxicity 3
		1st course				2nd course				
		DC	AT	Dose	# of Fractions	DC	AT			
		(× 10^7^)	(× 10^7^)	(Gy)		(× 10^7^)	(× 10^7^)			
Pancreatic	Lo × 1	2.1	34	60	15	2.4	25	458	RD	-
Cervical	Ln × 7	12	87	40	5	16	120	440	CR	-
Ovarian 4										
cycle 1	Lo × 1	2.5	33	50	10	2.1	34			-
cycle2	Lo × 2	6.7	76	35	5	3	76	415	CR	-
Breast	Ln × 2	2.7	5.7	40	4	3.5	6.5	377	RD	A
Uterine	Lo × 1	2.8	25	41	5	2.4	26	342	CR	B
Breast	Ln, Pl	1.4	34	40	4	1.3	33	317	CR	-
Colon	Lo × 2, Pe × 2	8	130	37	10	9.1	97	282	RD	-
Ovarian	Ln × 3	2.9	69	40	5	4.9	47	275	RD	C
Lung	Lo × 2	7.3	96	50	5	4.5	76	260	CR	D
Breast	Ln × 1	2.4	33	50	9	1.5	29	238	CR	-
Prostate 4										
cycle 1	B, Lo × 1	5.2	93	45	5	5.3	100			-
cycle 2	B × 13	12	210	40	5	25	310	214	RD	-
Gastric	Lo × 1, Pe	1.2	4.4	45	9	1.5	110	174	CR	-
Breast	Lo × 4	4.1	54	42	10	5.3	36	173	CR	-
Gastric	Ln × 6	17	160	42	10	14	140	149	CR	-
Lymphoma 4										
cycle 1	Ln × 9	8.3	170	30	5	6.7	220			-
cycle 2	Ln × 8	13	120	30	5	10	150	148	CR	-
Anal	Pe × 6, B × 2	18	120	40	10	14	100	141	CR	-
Renal	B × 2	5.7	100	41	3	3.6	73	138	RD	-
Ileocecal	Lo, Pe × 3	10	140	49	10	11	140	134	RD	-
Lung	Lo × 1, Ln × 2	18	110	50	5	18	110	130	RD	-
Lung	Lu × 2	3.3	80	50	5	2.5	83	123	CR	-
Cervical	Ln × 5	4.7	100	42	6	5.4	74	115	CR	-
Colon	Lu, Ln × 1, Ub	4.5	33	31	5	3.4	40	-	PD	E
Breast	Lo × 2, Ln × 1	9	49	45	5	8.9	49	-	PD	A
Pancreatic	Lo × 1	3.2	62	49	10	6.1	68	-	PD	-
Breast	B, Lu, Ln, Pl × 4	5.3	76	40	5	8.1	89	-	PD	-
Lung	B × 1, Lo × 2, Ln × 2	8.4	130	40	5	6.7	150	-	PD	F

1 Sites of tumor recurrence and treatment: Ab, abdominal wall; B, bone; Ln, lymph nodes; Lo, local; Lu, lung; Pe, peritoneum; Pl, pleura; Ub, urinary bladder;

2 Treatment response: CR, complete response, no disease at time of follow-up; RD, initial CR at treated sites but developed recurrent disease at distant sites at follow-up; PD, progressive disease;

3 Patient had radiation-associated toxicity (A, radiodermatitis; B, proctitis; C, peripheral nerve palsy; D, pneumonitis; E, peritonitis; F, pleuritis);

4 Patient underwent two cycles of immunotherapy.

**Table 4.** Serum tumor marker levels during treatment.

**Cancer**	**Tumor Marker**	**Pre**	**Post-1**	**Post-2**
Cervical	SCC(<1.5)	34.7	11.4	0.5
Ovarian				
cycle 1	CA125(<35)			
cycle 2	CA125(<35)	60 16.8	8.4 6.0	13.2 5.9
Ovarian	CA125(<35)	297	299	69
Breast	NCC-ST-439(<7)	31.5	14.5	1.9
Prostate				
cycle 1	PSA(<4.0)	25.8	14.0	
cycle 2	PSA(<4.0)	68.3	15.9	12.6 3.3
Gastric	CEA(<5.0)	35.4	41.2	2.3
Anal	SCC(<1.5)	80.6	41.4	0.5
Cervical	CA19-9(<37)	320	80	58
Colon	CEA(<5.0)	15.2	11.6	3.5
Pancreatic	CA19-9 (<37)	2300	3100	296.9
Breast	NCC-ST-439(<7)	321	425	196

Sample collection: Pre, at enrollment; Post-1, at evaluation shortly after 2nd iDC injection and AT infusion; Post-2, at last evaluation.

**Table 5.** Cytokine response of PBMC from a breast cancer patient stimulated with lysates of the breast cancer cell line, MCF7.

**Timepoint**	**Cytokines (pg/mL)**			

	**IL-10**	**IL-1β**	**IL-6**	**TNFα**
Enrollment	13	7	237	13
After 1st course 1	16	10	592	21
After 2nd course 2	9	3	145	8
25 days post-treatment	182	100	2910	260

1 First course = intratumoral iDC injection and AT infusion before IMRT;

2 Second course = intratumoral iDC injection and AT infusion after IMRT
